# Association of prehospital advanced airway and epinephrine with survival in patients with out-of-hospital cardiac arrest

**DOI:** 10.1038/s41598-023-44991-x

**Published:** 2023-10-19

**Authors:** Sejoong Ahn, Bo-Yeong Jin, Hanjin Cho, Sungwoo Moon, Young-Duck Cho, Jong-Hak Park

**Affiliations:** 1 Department of Emergency Medicine, Korea University Ansan Hospital, 123, Jeokgeum-ro, Danwon-gu, Ansan-si, Gyeonggi-do 15355 Republic of Korea; 2 Department of Biomedical Sciences, Seoul National University College of Medicine, Seoul, Republic of Korea; 3 Department of Emergency Medicine, Korea University Guro Hospital, Seoul, Republic of Korea

**Keywords:** Cardiovascular diseases, Cardiology, Medical research

## Abstract

Survival benefits of prehospital advanced airway and epinephrine in out-of-hospital cardiac arrest (OHCA) patients are controversial, but few studies evaluated this together. This study evaluated association of prehospital advanced airway and epinephrine with survival outcomes in OHCA patients. This was observational study using a prospective multicentre KoCARC registry. Adult OHCA patients between October 2015 and December 2021 were included. The variables of interest were prehospital managements, which was classified into basic life support (BLS)-only, BLS + advanced airway, and BLS + advanced airway + epinephrine. In total, 8217 patients were included in analysis. Survival to discharge and good neurological outcomes were lowest in the BLS + advanced airway + epinephrine group (22.1% in BLS-only vs 13.2% in BLS + advanced airway vs 7.5% in BLS + advanced airway + epinephrine, *P* < 0.001 and 17.1% in BLS-only vs 9.2% in BLS + advanced airway vs 4.3% in BLS + advanced airway + epinephrine, *P* < 0.001, respectively). BLS + advanced airway + epinephrine group was less likely to survive to discharge and have good neurological outcomes (aOR 0.39, 95% CI 0.28–0.55, *P* < 0.001 and aOR 0.33, 95% CI 0.21–0.51, *P* < 0.001, respectively) than BLS-only group after adjusting for potential confounders. In prehospital settings with intermediate EMS providers and prehospital advanced airway insertion is performed followed by epinephrine administration, prehospital management with BLS + advanced airway + epinephrine in OHCA patients was associated with lower survival to discharge rate compared to BLS-only.

## Introduction

Out-of-hospital cardiac arrest (OHCA) occurs annually in approximately 356,000 patients in the United States^1^ and 275,000 patients in Europe^2^, and mortality remains high. High-quality cardiopulmonary resuscitation (CPR) is important for patients with OHCA^3^. Advanced airway management and epinephrine administration with high-quality CPR concurrently can be performed in the prehospital phase^4^.

There are controversies regarding the survival benefits of prehospital advanced airway management and the administration of epinephrine. Some studies report survival benefits^5,6^, while others report poor survival outcomes from prehospital advanced airway management^7–10^ or no survival benefit at all^11–13^. Studies on prehospital epinephrine administration report good survival outcomes^14^, poor survival outcomes^15–17^ and no benefit at all^18,19^. These conflicting results could be due to differences in the setting of emergency medical service (EMS) systems or differences in the performance of EMS providers in those studies.

Furthermore, prehospital advanced airway management and epinephrine administration can be performed simultaneously. However, only a few previous studies have evaluated prehospital advanced airway management and epinephrine administration together^20,21^. Therefore, this study aimed to evaluate the association between prehospital advanced airway management and prehospital administration of epinephrine performed by intermediate-level emergency medical technicians (EMT) with survival outcomes in OHCA. We hypothesised that survival would be lower in the group with prehospital advanced airway management and prehospital administration of epinephrine in addition to basic life support, than in basic life support only.

## Methods

### Study design and setting

This study retrospectively analysed a prospective multicentre OHCA registry, the Korean Cardiac Arrest Resuscitation Consortium (KoCARC) registry. The KoCARC registry enrolled patients with OHCA with resuscitation attempts and presumed medical etiology who were transported by EMS to the Emergency Department of participating hospitals^22^. The exclusion criteria for the KoCARC registry were OHCA due to nonmedical etiology, hospice care, terminal illness, pregnancy, and do not resuscitate order. The KoCARC registry was registered with ClinicalTrials.gov (NCT03222999) and approved by the Institutional Review Boards of the participating hospitals.

This study was conducted in accordance with the principles of the Declaration of Helsinki. The Institutional Review Board of Korea University Ansan Hospital approved this study and waived the requirement for informed consent due to the nature of retrospective observational study (2022AS0309).

In South Korea, the National Fire Agency operates the EMS system. For OHCA cases, ambulances with 2–3 EMS providers are dispatched from regional EMS agencies belonging to fire departments. EMS providers consist of level-1 trained EMT (equivalent to EMT-intermediate in the North American EMS), level-2 EMT (equivalent to EMT-basic), or nurses, and provide basic to intermediate levels of service. Advanced airway insertion and epinephrine use before hospitalisation at the scene are only permitted for level-1 EMT. EMS providers at the scene are recommended to provide BLS for 6 min. Subsequently, the decision on advanced airway management and the administration of epinephrine is made at the discretion of the EMS providers. In most cases, a supraglottic airway is used for advanced airway management. The administration of epinephrine is generally not allowed for EMS providers; however, in cases where the EMS providers have received additional training for administration of epinephrine and are under the direct supervision of medical supervision of EMS physicians via telephone, epinephrine is administered intravenously. Intraosseous access is not permitted for EMT. Declaring death at the scene is prohibited, except for patients with obvious signs of death.

### Study population and data extraction

Adult patients (age ≥ 19 and < 80 years) with OHCA from October 2015 to December 2021 were included in this study. Patients with unknown prehospital management, unknown Utstein variables, unknown or extreme values of prehospital time, and those with OHCA witnessed during transport by EMS were excluded. Patients with a scene time of less than 6 min were excluded, because a short scene time may reflect the achievement of the return of spontaneous circulation (ROSC) prior to any resuscitation efforts and prehospital management by EMT, which may have biased our study.

The following data were extracted from the KoCARC registry: prehospital management, such as prehospital advanced airway management, prehospital administration of epinephrine, sex, age, witness status, location of cardiac arrest, bystander CPR, initial cardiac arrest rhythm, prehospital defibrillation, prehospital defibrillation time, total prehospital time, call to patient contact time, scene time, transport time, prehospital ROSC, survival to discharge and neurological outcome at discharge. A good neurological outcome was defined as a cerebral performance category score of 1 or 2.

### Variable of interest

The variable of interest was prehospital management performed by EMT. Prehospital management was classified as basic life support only (BLS only), basic life support followed by prehospital advanced airway insertion (BLS + advanced airway) and basic life support followed by prehospital advanced airway insertion and administration of epinephrine use (BLS + advanced airway + epinephrine).

### Outcomes

The primary outcome was survival to discharge. Secondary outcome was a good neurological outcome at discharge.

### Statistical analysis

Continuous and normally distributed variables were expressed as means and standard deviations and compared using the Student’s t test. Continuous and nonnormally distributed variables were expressed as medians and interquartile ranges, and compared using the Mann–Whitney U test. Categorical variables were expressed as numbers and percentages and compared using chi-square or Fisher exact tests. Bonferroni corrections were used in the post hoc analysis.

Multivariable logistic regression analyses were performed to identify independent associations between prehospital management level and outcomes. Sex, age, witnessed or not, location of cardiac arrest, bystander CPR, initial cardiac arrest rhythm, prehospital defibrillation, response time, scene time, transport time, and prehospital ROSC were used for adjustment. The Hosmer–Lemeshow test was used to evaluate the goodness of fit of the models.

Restricted cubic spline analysis was performed to evaluate the nonlinear association between the level of prehospital management and survival to discharge according to scene time. A restricted cubic spline curve with five knots was used after adjustment for the variables used in the multivariable logistic regression analysis.

Subgroup analysis was performed according to the initial cardiac arrest rhythm and the witness status. In addition, a subgroup of patients who may be candidates for extracorporeal membrane oxygenation-assisted CPR (ECPR) (defined as patients who were witnessed, had an initial shockable rhythm, and were aged < 75 years) was analysed^23,24^. Sensitivity analyses were performed including cases with scene time < 6 min and including cases with age > 80 years.

Statistical significance was set less than 0.05. Statistical analyses were performed using R version 4.0.2 (R Foundation for Statistical Computing, Vienna, Austria).

### Ethics approval

The institutional review board approved this study and waived the requirement for informed consent (2022AS0309).

## Results

**Figure 1:**
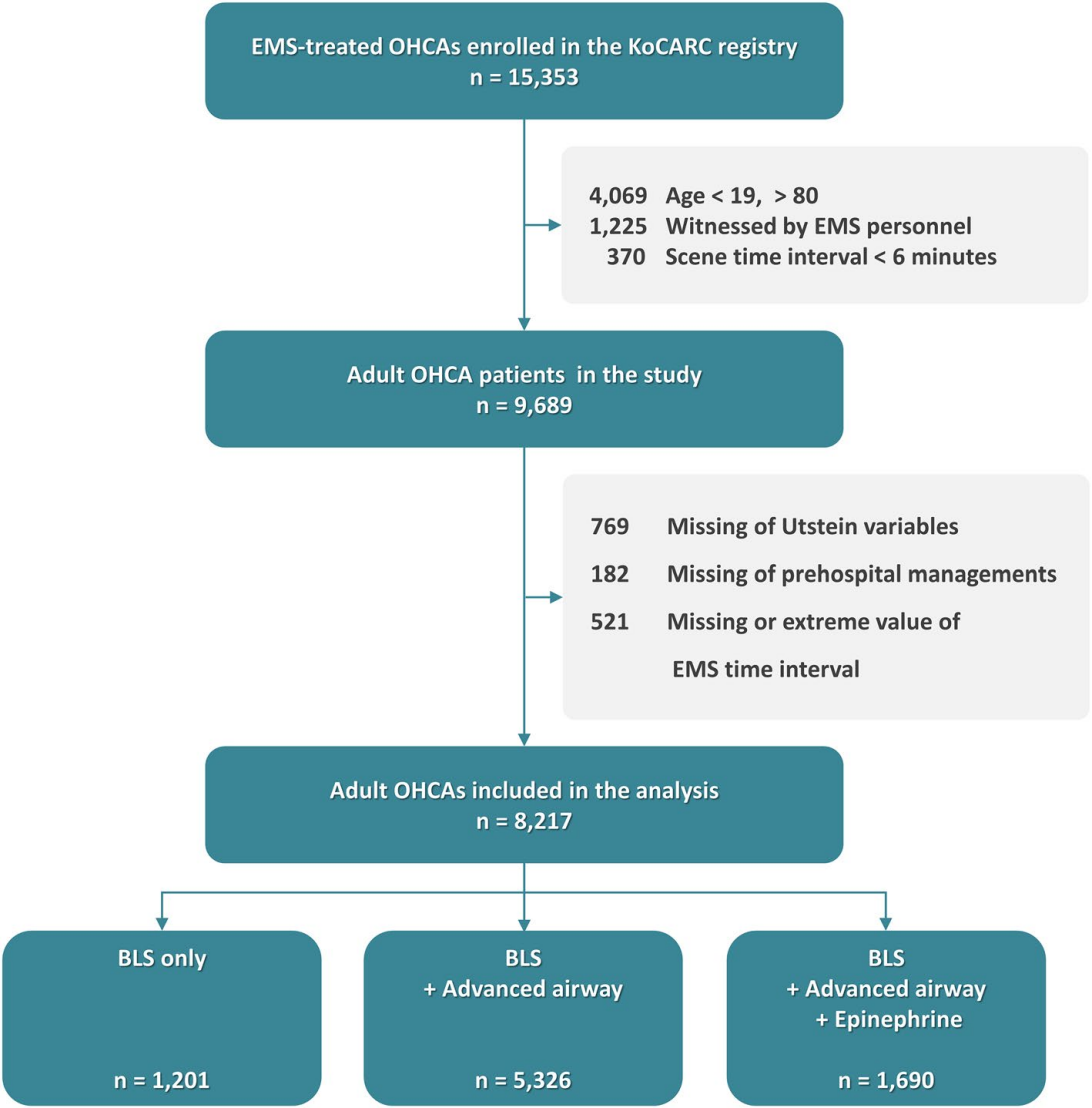
Flow chart of the study population. OHCA, out-of-hospital cardiac arrest; KoCARC, Korean Cardiac Arrest Resuscitation Consortium; EMS, emergency medical services.

Between October 2015 and December 2021, 15,353 patients with OHCA were enrolled in the KoCARC registry. Among them, 7136 patients were excluded due to age (n = 4069), unknown prehospital management (n = 182), unknown Utstein variables (n = 769), EMS witnessed cardiac arrest during transport (n = 1225), unknown/extreme values of prehospital time (n = 521), and scene time less than 6 min (n = 370). Finally, 8,217 patients were included in the analysis (Fig. 1). The mean and median age of the study population was 62.8 ± 13.4 years and 65 [54–74] years. Among the study population, 71.4% were men, 28.6% were women, 13.3% survived to discharge, and 9.4% had good neurological outcomes.

Table 1 shows the baseline characteristics and outcomes according to prehospital management level. Age was older in the BLS + advanced airway group (*P* = 0.001), witnessed cardiac arrest was most frequent in the BLS only group (*P* < 0.001), and bystander CPR was performed most frequently in the BLS + advanced airway + epinephrine group (*P* < 0.001). Initial shockable rhythm was frequent in the BLS only group (*P* < 0.001), and prehospital defibrillation was least frequently performed in the BLS + advanced airway group. Total prehospital time, response time, and scene time were significantly longer in the BLS + advanced airway + epinephrine group than in the BLS only and BLS + advanced airway groups (total prehospital time (min):28 [23–35] in BLS only vs. 29 [24–34] in BLS + advanced airway vs. 37 [31–43] in BLS + advanced airway + epinephrine, *P* < 0.001; response time (min): 8 [7–11] in BLS only vs. 8 [7–11] in BLS + advanced airway vs. 9 [7–11] in BLS + advanced airway + epinephrine, *P* < 0.001; scene time (min):11 [9–15] in BLS only vs. 13 [10–16] in BLS + advanced airway vs. 19 [15–24] in BLS + advanced airway + epinephrine, *P* < 0.001). I-gel was predominantly used for prehospital advanced airway management in BLS + advanced airway group and BLS + advanced airway + epinephrine group.

**Table 1:** Baseline characteristics and outcomes of study populations.

	Overall (n = 8217)	BLS only (n = 1201)	BLS + advanced airway (n = 5326)	BLS + advanced airway + epinephrine (n = 1690)	P-value
Age, year	65 [54–74]	64 [53–73]	66 [55–75]	64 [54–73]	0.001^a,c^
Sex, female, n (%)	2349 (28.6%)	358 (29.8%)	1572 (29.5%)	419 (24.8%)	0.001^b,c^
Witnessed arrest, n (%)	4270 (52.0%)	741 (61.7%)	2964 (55.7%)	865 (51.2%)	< 0.001^a,b,c^
Public place, n (%)	1713 (20.8%)	267 (22.2%)	1082 (20.3%)	364 (21.5%)	0.247
Bystander CPR, n (%)	4876 (59.3%)	627 (52.2%)	3161 (59.4%)	1088 (64.4%)	< 0.001^a,b,c^
Initial shockable rhythm, n (%)	1893 (23.0%)	332 (27.6%)	1171 (22.0%)	390 (23.1%)	< 0.001^a^
EMS time interval, minute					
Response time	8 [7–11]	8 [7–11]	8 [7–11]	9 [7–11]	< 0.001^b,c^
Scene time	14 [10–17]	11 [9–15]	13 [10–16]	19 [15–24]	< 0.001^a,b,c^
Transport time	7 [5–10]	7 [5–11]	7 [5–9]	7 [5–10]	0.004^a^
Total pre-hospital time	30 [25–36]	28 [23–35]	29 [24–34]	37 [31–43]	< 0.001^b,c^
EMS management					
Defibrillation, n (%)	2565 (31.2%)	426 (35.5%)	1573 (29.5%)	566 (33.5%)	< 0.001^a,c^
Advanced airway, n (%)	7016 (85.4%)	0 (0%)	5326 (100%)	1690 (100%)	
Endotracheal intubation, n (%)	920 (11.2%)	0 (0%)	665 (12.5%)	255 (15.1%)	< 0.001^c^
SGA, n (%)	6096 (74.2%)	0 (0%)	4661 (87.5%)	1435 (84.9%)	< 0.001^c^
Combitube, n (%)	1 (0%)	0 (0%)	1 (0%)	0 (0%)	
King airway, n (%)	53 (0.6%)	0 (0%)	47 (0.9%)	6 (0.4%)	
LMA, n (%)	58 (0.7%)	0 (0%)	42 (0.8%)	16 (0.9%)	
I-gel, n (%)	5984 (72.8%)	0 (0%)	4571 (85.8%)	1413 (83.6%)	
Epinephrine, n (%)	1690 (20.6%)	0 (0%)	0 (0%)	1690 (100%)	
Pre-hospital ROSC, n (%)	1217 (14.8%)	262 (21.8%)	661 (12.4%)	294 (17.4%)	< 0.001^a,b,c^
Time to prehospital ROSC, minute*	18 [13–25]	14 [11–20]	17 [13–23]	27 [21–34]	< 0.001^a,b,c^
Time to ROSC, minute**	34 [21–44]	29 [15–41]	34 [22–44]	36 [26–48]	< 0.001^a,b,c^
Survival outcome					
Survival to discharge, n (%)	1095 (13.3%)	266 (22.1%)	703 (13.2%)	126 (7.5%)	< 0.001^a,b,c^
Good neurologic recovery, n (%)	770 (9.4%)	205 (17.1%)	492 (9.2%)	73 (4.3%)	< 0.001^a,b,c^

Data are expressed as median [interquartile range] or number (percentage) as appropriate.

CPR, cardiopulmonary resuscitation; EMS, emergency medical service; SGA, supraglottic airway; ROSC, return of spontaneous circulation.

*n = 935 (n = 188 for BLS only, n = 521 for BLS + advanced airway group, n = 226 for BLS + advanced airway + epinephrine groups). The variable was evaluated for those without missing time variables.

*﻿*n = 2350 (n = 404 for BLS only, n = 1511 for BLS + advanced airway group, n = 435 for BLS + advanced airway + epinephrine groups). ROSC included prehospital ROSC and in-hospital ROSC. The variable was evaluated for those without missing time variables.

^a^Significant difference between the BLS only and BLS + advanced airway groups after Bonferroni correction in post hoc analysis.

^b^Significant difference between the BLS only and BLS + advanced airway + epinephrine groups after Bonferroni correction in post-hoc analysis.

^c^Significant difference between the BLS + advanced airway and BLS + advanced airway + epinephrine groups after Bonferroni correction in post hoc analysis.

The rate of prehospital ROSC was lowest in the BLS + advanced airway group (21.8% in BLS only vs 12.4% in BLS + advanced airway vs 17.4% in BLS + advanced airway + epinephrine, *P* < 0.001). Among those who achieved prehospital ROSC, time to prehospital ROSC was significantly longer in the BLS + advanced airway + epinephrine group than in the BLS only and BLS + advanced airway groups (14 min in BLS only vs 17 min in BLS + advanced airway vs 27 min in BLS + advanced airway + epinephrine, *P* < 0.001).

The rate of survival to discharge and good neurological outcomes were lowest in the BLS + advanced airway + epinephrine group and were significantly different among the groups (survival to discharge: 22.1% in BLS only vs 13.2% in BLS + advanced airway vs 7.5% in BLS + advanced airway + epinephrine, *P* < 0.001; good neurologic outcome: 17.1% in BLS only vs 9.2% in BLS + advanced airway vs 4.3% in BLS + advanced airway + epinephrine, *P* < 0.001; Table 1).

### Comparison of scene arrival to first defibrillation time in patients with initial shockable rhythm by level of prehospital management

Scene arrival to first defibrillation time was longer in the BLS + advanced airway + epinephrine group than in the BLS only group and the BLS + advanced airway group (2.1 ± 3.2 min in the BLS only vs 2.0 ± 2.3 min in the BLS + advanced airway vs 2.7 ± 2.3 min in the BLS + advanced airway + epinephrine, *P* < 0.001; Table 2).

**Table 2:** Scene arrival to first defibrillation time in patients with initial shockable rhythm according to the level of prehospital management.

Variables	BLS only (n = 302)	BLS + advanced airway (n = 1133)	BLS + advanced airway + epinephrine (n = 363)	P-value
Scene arrival to first defibrillation time	1.5 [1–2]	1 [1–2]	2 [1–4]	< 0.001^b,c^
	2.1 ± 3.2	2.0 ± 2.3	2.7 ± 2.3	< 0.001^b,c^

Data are expressed as median [interquartile range], or mean ± standard deviation.

^a^Significant difference between the BLS only and BLS + advanced airway groups after Bonferroni correction in post hoc analysis.

^b^Significant difference between the BLS only and BLS + advanced airway + epinephrine groups after Bonferroni correction in post-hoc analysis.

^c^Significant difference between the BLS + advanced airway and BLS + advanced airway + epinephrine groups after Bonferroni correction in post hoc analysis.

### Multivariable logistic regression analysis

**Figure 2:**
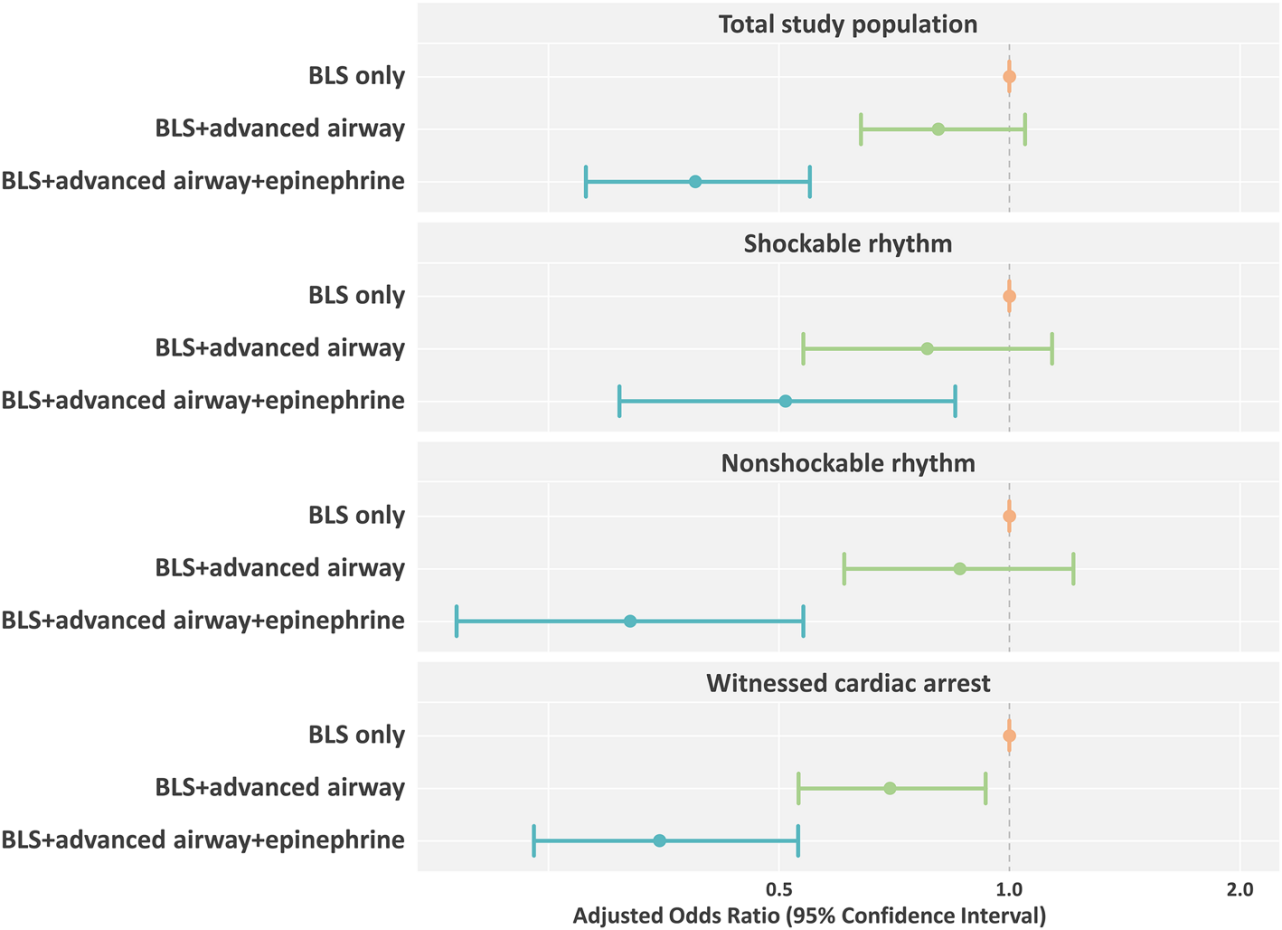
Multivariable logistic regression analysis on survival to discharge. Multivariate logistic regression analysis was performed after adjusting for sex, age, witnessed status, place of cardiac arrest, bystander CPR, initial cardiac arrest rhythm, prehospital defibrillation, response time, scene time, transport time, and prehospital ROSC. CPR, cardiopulmonary resuscitation; ROSC, return of spontaneous circulation.

**Figure 3:**
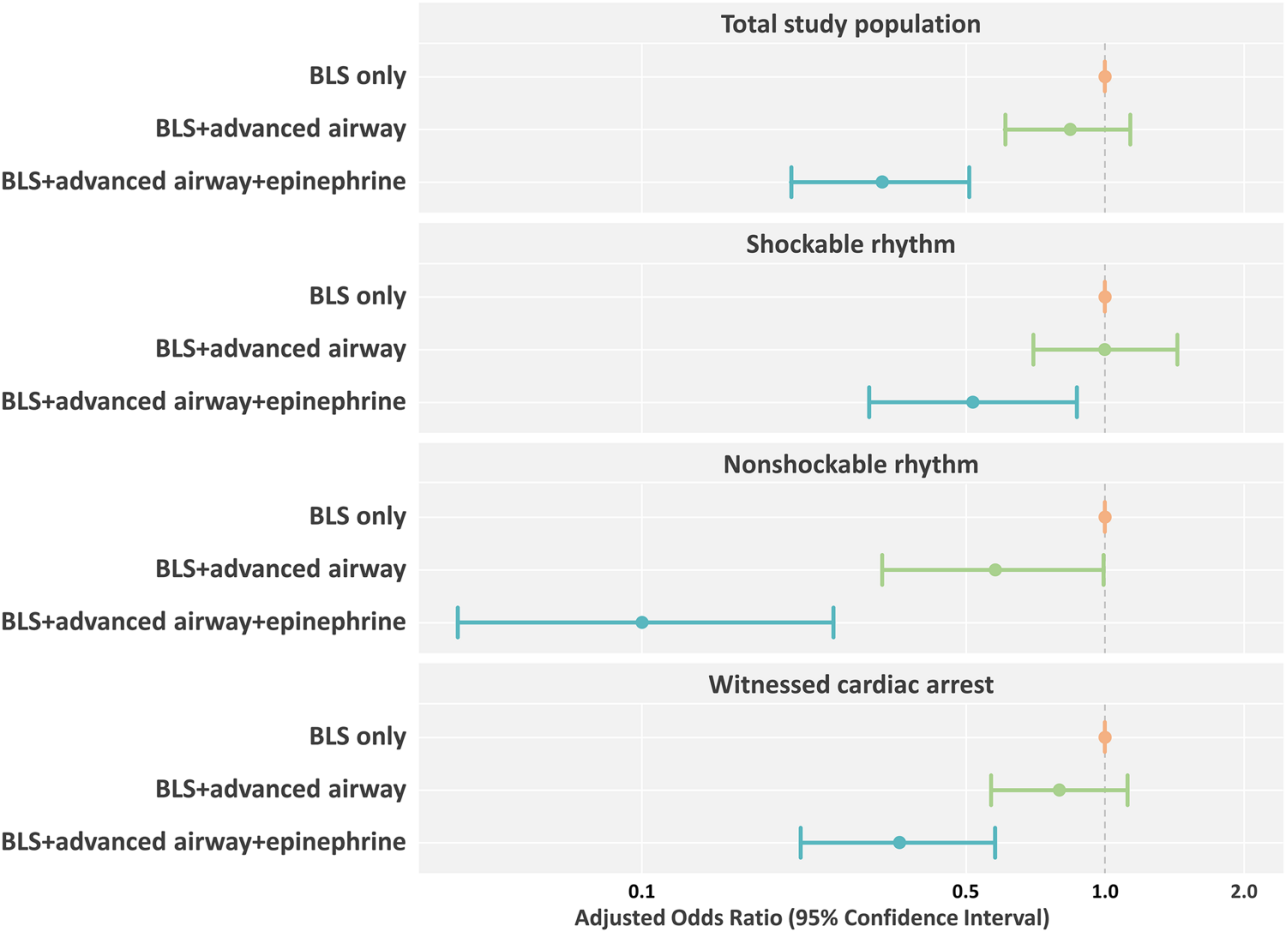
Multivariable logistic regression analysis on good neurological outcome. Multivariate logistic regression analysis was performed after adjusting for sex, age, witnessed status, place of cardiac arrest, bystander CPR, initial cardiac arrest rhythm, prehospital defibrillation, response time, scene time, transport time, and prehospital ROSC. CPR, cardiopulmonary resuscitation; ROSC, return of spontaneous circulation.

The BLS + advanced airway + epinephrine group was less likely to survive to discharge and less likely to have good neurological outcomes (adjusted odd’s ratio (aOR) 0.39, 95% confidence interval (CI) 0.28–0.55, *P* < 0.001 and aOR 0.33, 95% CI 0.21–0.51, *P* < 0.001, respectively) than the BLS only group after adjusting for sex, age, witnessed status, place of cardiac arrest, bystander CPR, initial cardiac arrest rhythm, prehospital defibrillation, response time, scene time, transport time, and prehospital ROSC (Figs. 2 and 3).

All models showed a good fit in the Hosmer–Lemeshow test (all *P* > 0.05).

### Restricted cubic spline analysis by level of prehospital management according to scene time

**Figure 4:**
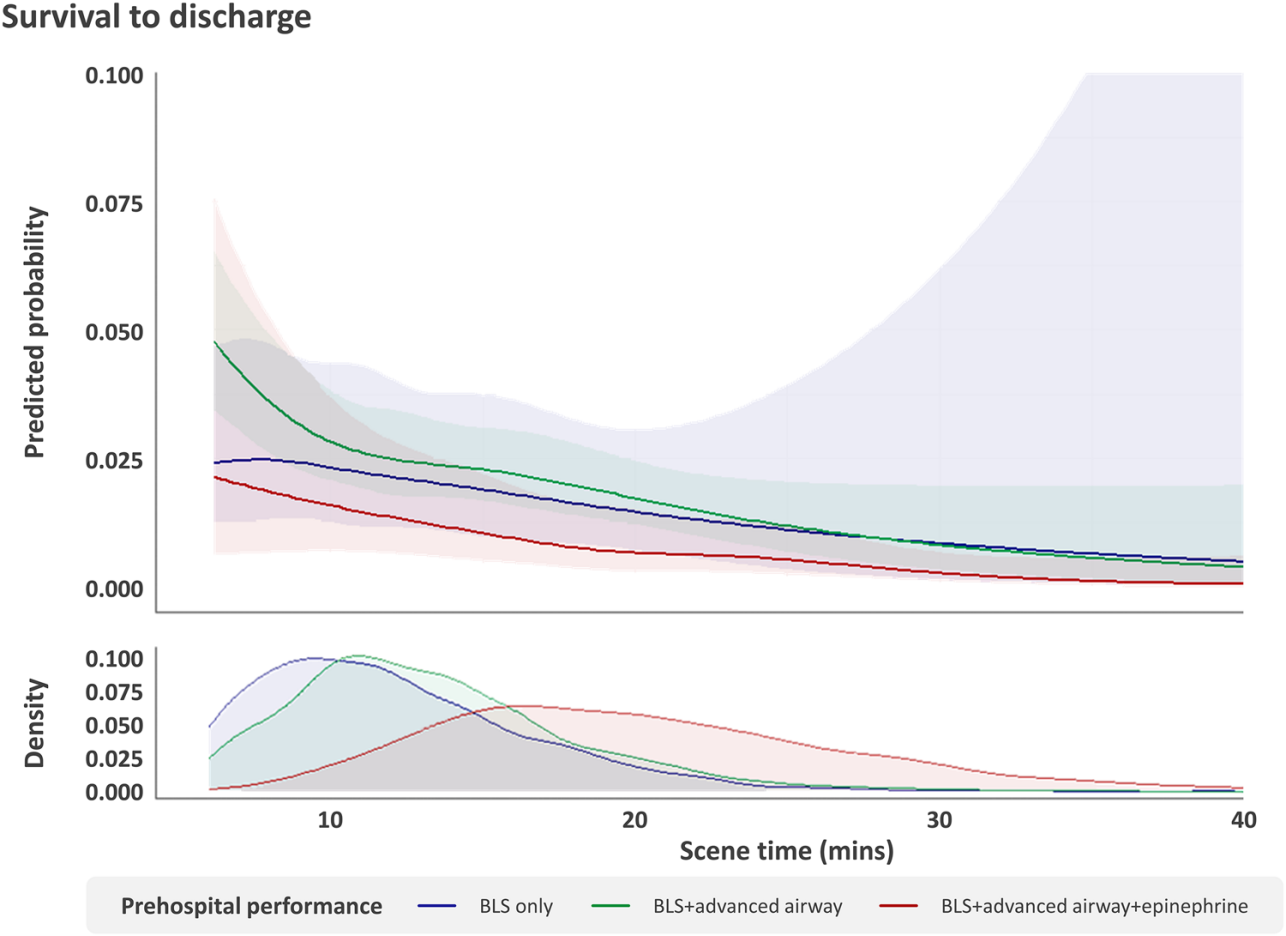
Association between the level of prehospital management and survival to discharge according to scene time. The restricted cubic spline curve shows an adjusted association between the level of prehospital management (BLS only (navy), BLS + advanced airway (green), and BLS + advanced airway + epinephrine (red)) and the predicted probability of survival to discharge according to scene time. The shaded area indicates the 95% confidence interval of the predicted probability. The model was adjusted for sex, age, witnessed status, location of cardiac arrest, bystander CPR, initial cardiac arrest rhythm, prehospital defibrillation, response time, scene time, transport time, and prehospital ROSC. CPR, cardiopulmonary resuscitation; ROSC, return of spontaneous circulation.

The restricted cubic spline curve according to scene time shows a decreasing adjusted predicted probability of survival to discharge as scene time increased in all groups. The adjusted predicted probability was lower in the BLS + advanced airway + epinephrine group than in the other groups in all sections of the scene time. The adjusted predicted probability peaked in the BLS + advanced airway group within a scene time < 10 min and became comparable to the BLS only group at the rest of the scene time (Fig. 4).

### Subgroup analysis

The BLS + advanced airway + epinephrine group was less likely to survive to discharge in all subgroups (aOR 0.51, 95% CI 0.31–0.85, *P* = 0.009 in the subgroup with a shockable rhythm; aOR 0.32, 95% CI 0.19–0.54, *P* < 0.001 in the subgroup with a non-shockable rhythm; aOR 0.35, 95% CI 0.24–0.53, *P* < 0.001 in the subgroup with witnessed cardiac arrest; Fig. 2). The BLS + advanced airway + epinephrine group was less likely to have good neurological outcomes in all subgroups (aOR 0.52, 95% CI 0.31–0.87, *P* = 0.012 in the subgroup with a shockable rhythm; aOR 0.10, 95% CI 0.04–0.26, *P* < 0.001 in the subgroup with a nonshockable rhythm; aOR 0.36, 95% CI 0.22–0.58, *P* < 0.001 in the subgroup with a witnessed cardiac arrest; Fig. 3).

The BLS + advanced airway + epinephrine group was less likely to survive to discharge and less likely to have good neurologic outcomes in the subgroup with possible candidates for ECPR (aOR 0.39, 95% CI 0.22–0.71, *P* = 0.002 and 0.55, 95% CI 0.31–0.99, *P* = 0.045, respectively; supplementary Table 1).

### Sensitivity analysis

The proportions of initial shockable rhythm and defibrillation in cases with scene time < 6 min were comparable between groups (supplementary Table 2). There were 8,587 patients with cases including a scene time of < 6 min. The BLS + advanced airway + epinephrine group was less likely to survive to discharge and less likely to have good neurological outcome (supplementary Table 3).

There were 11,904 patients with cases including those aged > 80 years. The BLS + advanced airway + epinephrine group was less likely to survive to discharge and less likely to have the good neurological outcome (supplementary Table 4).

## Discussion

In patients with OHCA, prehospital management with BLS with advanced airway insertion and prehospital epinephrine administration was associated with decreased survival to discharge rate and decreased good neurological outcome rate than prehospital management with BLS alone. These associations were maintained in the various subgroups. The predicted probability of survival to discharge was lower in the prehospital management with BLS with advanced airway insertion and prehospital epinephrine use group than in the other groups in all sections of scene time. Survival results were comparable between prehospital management with BLS with advanced airway insertion and the BLS-only groups.

The strengths of our study are that it evaluated two prehospital managements that could be performed simultaneously or independently, and evaluated their associations with survival outcomes using a prospective multicentre registry. In addition, we performed various subgroup analyses, including subgroups of possible candidates for ECPR. Additionally, we evaluated the adjusted predicted probability of survival of the groups according to the scene time. Our results may provide insight into the controversies surrounding prehospital management.

Prehospital management could alter the quality of chest compressions during the prehospital phase. Previous studies reported that prehospital management was associated with interruption of chest compression^25,26^. The administration of prehospital epinephrine after insertion of the prehospital advanced airway requires considerable effort by 2–3 EMS providers to maintain CPR when compared to BLS alone or BLS with prehospital advanced airway management. Although the quality of CPR by EMS providers was not collected in our study, a significantly longer scene time and a significantly longer first defibrillation time in BLS with prehospital advanced airway management and epinephrine group may reflect poor overall quality of prehospital CPR. A delayed first defibrillation time can lead to poor outcomes in patients with shockable rhythm. Additionally, when we assessed the initial vital signs of successfully resuscitated individuals, we observed that the BLS only group had the highest initial blood pressure, whereas the BLS + advanced airway + epinephrine group had the lowest (supplementary Table 5). The difference between groups was significant. Furthermore, initial Glasgow Coma Scale score was significantly higher in BLS only group than in other groups, and the presence of pupillary light reflex, corneal reflex, and self-respiration were more frequent in BLS only group (supplementary Table 5). This may indicate an overall higher quality of CPR in BLS only group compared to other groups. Considering the results of defibrillation time, initial vital signs, and mental status between the groups, transporting patients with OHCA without administering epinephrine in the prehospital phase might be better than trying prehospital administration of epinephrine at the scene in prehospital settings with EMS providers with intermediate EMT.

The time to the first prehospital administration of epinephrine may be important. Previous studies reported that prehospital administration of epinephrine in 20 min was associated with survival and good neurological outcomes^27,28^. However, delayed prehospital epinephrine administration is associated with poor outcomes^29^. In our study, there were 723 patients for whom the first prehospital epinephrine time was available. The mean and median time to the first prehospital epinephrine was 22.2 ± 8.4 min and 20^16–26^ mins. Of these patients, 55.7% received the first prehospital epinephrine after 20 min. EMS providers with intermediate EMT may be associated with a delay in the time to first prehospital epinephrine administration compared to EMS providers with paramedics or doctors, and this delay in the first prehospital epinephrine administration may lead to poor survival outcomes.

Increased scene time was associated with poor outcomes in all groups, according to the restricted cubic spline curve. The predicted probability of the group with prehospital advanced airway insertion and prehospital epinephrine use was the lowest in all section of scene time. It may be better to transport patients with OHCA without prehospital epinephrine use to gain the benefit of reduced scene time. On the other hand, to maximise the positive effect of prehospital advanced airway insertion, attempts need to be made if it is likely to be successful within 10 min of scene time. If this is not achievable, it may be better to transport OHCA patients without advanced airways to reduce the overall scene time.

There may be a combined effect between prehospital advanced airway management and prehospital adrenaline administration. Most patients received prehospital epinephrine in previous studies that evaluated the effect of prehospital advanced airway^7,9,11^. Few previous studies have evaluated prehospital advanced airway and epinephrine use together^20,21^. A previous study with paramedics as EMS providers supports our results^20^. The outcome of the previous study was poorer in the BLS with the prehospital advanced airway and prehospital epinephrine group than in the BLS only group. On the contrary, in a previous study with physicians as EMS providers, the outcome was comparable between the groups^21^. More advanced EMS providers, such as physicians, might be needed to prevent the adverse effects of BLS with prehospital advanced airway and epinephrine administration.

In the subgroup that may benefit from ECPR^23,24^, BLS with prehospital advanced airway and prehospital administration of epinephrine was associated with poor outcomes. In cases of ECPR, the time from cardiac arrest to the initiation of extracorporeal membrane oxygenation is critical^23,24^. Since the total prehospital time was significantly longer in the BLS with prehospital advanced airway and prehospital epinephrine group than in the other groups, the chance of receiving early ECPR could have decreased in the group with BLS with prehospital advanced airway and prehospital epinephrine. In cases with possible candidates for ECPR, not all prehospital managements, such as advanced airway and epinephrine, need to be performed to reduce the total prehospital time.

Our study had limitations. First, since this study was observational, there could be missing covariables, and we could only find associations. Second, this study was conducted in Korea with EMS providers with intermediate EMT. These results cannot be generalised to other countries with different prehospital settings. In prehospital setting in South Korea, the prehospital management of patients with OHCA is primarily based on BLS. Through direct medical supervision via telephone, EMS provider can further administer epinephrine. Consequently, there may be limitations in the nature of retrospective study due to potential selection bias in the discretion of EMS providers. In addition, advanced airway insertion (mostly using I-gel) is performed, followed by epinephrine. On the other hand, most patients in previous studies that evaluated the effect of prehospital advanced airway received prehospital epinephrine^7,9,11^. We evaluated prehospital advanced airway and prehospital epinephrine together. Considering our result and difference in order of prehospital management between setting in our study and setting in previous studies, our result may provide insight into prehospital management including order of prehospital management. Third, the quality of CPR provided by the EMS providers was not assessed. However, the overall quality of the CPR might be poor in BLS with prehospital advanced airway and prehospital epinephrine group, since the time lapse to first defibrillation was significantly longer and initial vital signs, initial mental status, and the result of neurologic examination in those who were successfully resuscitated were worse compared to other groups. Further studies are needed on the quality of prehospital CPR. Fourth, no data on comorbidities were collected. However, patients under hospice care or with terminal illnesses were excluded from our multicentre prospective registry. Fifth, we did not collect the do not resuscitate order or withdrawal of life-sustaining treatment after hospital admission. Sixth, prehospital epinephrine was only administered intravenously because intraosseous approach was prohibited for EMTs in our country. Using intraosseous access might lead to faster administration of adrenaline compared to intravenous access. Further studies are warranted in EMS settings with intraosseous access.

## Conclusions

In prehospital settings with EMS providers with intermediate EMT and prehospital advanced airway insertion is performed followed by epinephrine administration, prehospital management with BLS with advanced airway insertion and prehospital administration of epinephrine in OHCA patients was associated with decreased survival to discharge rate and poor neurological outcome rate compared to prehospital management with BLS alone. The survival outcomes were comparable between the BLS with prehospital advanced airway insertion and BLS only groups.

### Supplementary Information

Supplementary Tables.

## Data Availability

The data generated and/or analysed during the current study are not publicly available due to their containing information that could compromise the privacy of research participants but are available from the corresponding author on reasonable request.
