# Supplementary material for: Association of prehospital advanced airway and epinephrine with survival in patients with out-of-hospital cardiac arrest
Source: Sci Rep. 2023 Oct 19;13:17836. doi: 10.1038/s41598-023-44991-x (PMC10587122; doi:10.1038/s41598-023-44991-x)
Supplement: Supplementary file 1 — Supplementary Tables. [file 41598_2023_44991_MOESM1_ESM.docx]

**Supplementary Table 1.** Multivariable logistic regression analysis of cases with possible ECPR candidates

|  | aOR | 95% CI | p-value |
| --- | --- | --- | --- |
| **Survival to discharge** |  |  |  |
| BLS only | 1 (reference) |  |  |
| BLS+advanced airway | 0.70 | 0.46–1.09 | 0.114 |
| BLS+advanced airway+epinephrine | 0.39 | 0.22–0.71 | 0.002 |
| **Good neurologic outcome** |  |  |  |
| BLS only | 1 (reference) |  |  |
| BLS+advanced airway | 0.94 | 0.63–1.42 | 0.786 |
| BLS+advanced airway+epinephrine | 0.55 | 0.31–0.99 | 0.045 |

Abbreviations: ECPR, extracorporeal membrane oxygenation assisted cardiopulmonary resuscitation; aOR, adjusted odds ratio; CI, confidence interval; BLS, basic life support; CPR, cardiopulmonary resuscitation; ROSC, return of spontaneous circulation.

**Supplementary Table 2.** Baseline characteristics of cases with scene time <6mins.

|  | Overall  (n = 370) | BLS only  (n = 157) | BLS  + advanced airway  (n = 206) | BLS  + advanced airway  + epinephrine  (n = 7) | P- value |
| --- | --- | --- | --- | --- | --- |
| Age, year | 62 [51–72] | 63 [51–73] | 62 [52–72] | 56 [50–63] | 0.288 |
| Sex, female, n (%) | 98 (26.5%) | 47 (29.9%) | 50 (24.3%) | 1 (14.3%) | 0.365 |
| Witnessed arrest, n (%) | 247 (66.8%) | 112 (71.3%) | 130 (63.1%) | 5 (71.4%) | 0.248 |
| Public place, n (%) | 145 (39.2%) | 56 (35.7%) | 87 (42.2%) | 2 (28.6%) | 0.377 |
| Bystander CPR, n (%) | 177 (47.8%) | 71 (45.2%) | 99 (48.1%) | 7 (100.0%) | 0.018^a^ |
| Initial shockable rhythm, n (%) | 110 (29.7%) | 47 (29.9%) | 60 (29.1%) | 3 (42.9%) | 0.735 |
| EMS time interval, minute |  |  |  |  |  |
| Response time | 8 [6–12] | 9 [7–12] | 15 [11–20] | 9 [6–12] | 0.099 |
| Scene time | 4 [3–5] | 4 [3–5] | 4 [3–5] | 4 [3–5] | 0.946 |
| Transport time | 7 [5–12] | 7 [5–13] | 8 [5–11] | 14 [8–16] | 0.410 |
| Total pre-hospital time | 21 [17–28] | 21 [17–29] | 21 [17–26] | 27 [26–37] | 0.178 |
| EMS Management |  |  |  |  |  |
| Defibrillation, n (%) | 130 (35.1%) | 52 (33.1%) | 75 (36.4%) | 3 (42.9%) | 0.737 |
| Advanced airway, n (%) | 213 (57.6%) | 0 (0%) | 206 (100%) | 7 (100%) |  |
| Epinephrine, n (%) | 7 (1.9%) | 0 (0%) | 0 (0%) | 7 (100%) |  |

Data are expressed as median [interquartile range] or number (percentage) as appropriate.

^a^ Significant difference between the BLS only and BLS+advanced airway+epinephrine groups after Bonferroni correction in post-hoc analysis

Abbreviations: CPR, cardiopulmonary resuscitation; EMS, emergency medical service; SGA, supraglottic airway; ROSC, return of spontaneous circulation.

**Supplementary Table 3.** Multivariable logistic regression analysis of cases, including scene time <6mins

|  | aOR | 95% CI | p-value |
| --- | --- | --- | --- |
| **Survival to discharge** |  |  |  |
| **Total** |  |  |  |
| BLS only | 1 (reference) |  |  |
| BLS+advanced airway | 0.90 | 0.71–1.13 | 0.371 |
| BLS+advanced airway+epinephrine | 0.43 | 0.31–0.60 | <0.001 |
| **Shockable** |  |  |  |
| BLS only | 1 (reference) |  |  |
| BLS+advanced airway | 0.89 | 0.63–1.25 | 0.491 |
| BLS+advanced airway+epinephrine | 0.53 | 0.33–0.86 | 0.010 |
| **Non-shockable** |  |  |  |
| BLS only | 1 (reference) |  |  |
| BLS+advanced airway | 0.95 | 0.69–1.30 | 0.739 |
| BLS+advanced airway+epinephrine | 0.38 | 0.23–0.63 | <0.001 |
| **Witnessed cardiac arrest** |  |  |  |
| BLS only | 1 (reference) |  |  |
| BLS+advanced airway | 0.77 | 0.59–1.00 | 0.053 |
| BLS+advanced airway+epinephrine | 0.39 | 0.26–0.57 | <0.001 |
| **Good neurologic outcome** |  |  |  |
| **Total** |  |  |  |
| BLS only | 1 (reference) |  |  |
| BLS+advanced airway | 0.94 | 0.71–1.25 | 0.662 |
| BLS+advanced airway+epinephrine | 0.36 | 0.24–0.55 | <0.001 |
| **Shockable** |  |  |  |
| BLS only | 1 (reference) |  |  |
| BLS+advanced airway | 1.11 | 0.80–1.55 | 0.524 |
| BLS+advanced airway+epinephrine | 0.55 | 0.34–0.90 | 0.018 |
| **Non-shockable** |  |  |  |
| BLS only | 1 (reference) |  |  |
| BLS+advanced airway | 0.67 | 0.40–1.12 | 0.126 |
| BLS+advanced airway+epinephrine | 0.12 | 0.05–0.30 | <0.001 |
| **Witnessed cardiac arrest** |  |  |  |
| BLS only | 1 (reference) |  |  |
| BLS+advanced airway | 0.90 | 0.66–1.22 | 0.488 |
| BLS+advanced airway+epinephrine | 0.39 | 0.24–0.62 | <0.001 |

Multivariate logistic regression analysis was performed after adjusting for sex, age, witnessed status, place of cardiac arrest, bystander CPR, initial cardiac arrest rhythm, prehospital defibrillation, response time, scene time, transport time, and prehospital ROSC.

Abbreviations: aOR, adjusted odds ratio; CI, confidence interval; BLS, basic life support; CPR, cardiopulmonary resuscitation; ROSC, return of spontaneous circulation.

**Supplementary Table 4.** Multivariable logistic regression analysis of cases including age >80 years.

|  | aOR | 95% CI | p-value |
| --- | --- | --- | --- |
| **Survival to discharge** |  |  |  |
| **Total** |  |  |  |
| BLS only | 1 (reference) |  |  |
| BLS+advanced airway | 0.85 | 0.67–1.08 | 0.180 |
| BLS+advanced airway+epinephrine | 0.43 | 0.31–0.59 | <0.001 |
| **Shockable** |  |  |  |
| BLS only | 1 (reference) |  |  |
| BLS+advanced airway | 0.80 | 0.56–1.14 | 0.217 |
| BLS+advanced airway+epinephrine | 0.54 | 0.33–0.88 | 0.013 |
| **Non-shockable** |  |  |  |
| BLS only | 1 (reference) |  |  |
| BLS+advanced airway | 0.91 | 0.66–1.25 | 0.558 |
| BLS+advanced airway+epinephrine | 0.38 | 0.24–0.62 | <0.001 |
| **Witnessed cardiac arrest** |  |  |  |
| BLS only | 1 (reference) |  |  |
| BLS+advanced airway | 0.75 | 0.58–0.98 | 0.037 |
| BLS+advanced airway+epinephrine | 0.40 | 0.27–0.59 | <0.001 |
| **Good neurologic outcome** |  |  |  |
| **Total** |  |  |  |
| BLS only | 1 (reference) |  |  |
| BLS+advanced airway | 0.85 | 0.63–1.15 | 0.300 |
| BLS+advanced airway+epinephrine | 0.33 | 0.22–0.51 | <0.001 |
| **Shockable** |  |  |  |
| BLS only | 1 (reference) |  |  |
| BLS+advanced airway | 0.99 | 0.70–1.41 | 0.978 |
| BLS+advanced airway+epinephrine | 0.51 | 0.31–0.85 | 0.009 |
| **Non-shockable** |  |  |  |
| BLS only | 1 (reference) |  |  |
| BLS+advanced airway | 0.63 | 0.37–1.07 | 0.089 |
| BLS+advanced airway+epinephrine | 0.11 | 0.05–0.27 | <0.001 |
| **Witnessed cardiac arrest** |  |  |  |
| BLS only | 1 (reference) |  |  |
| BLS+advanced airway | 0.83 | 0.60–1.15 | 0.255 |
| BLS+advanced airway+epinephrine | 0.37 | 0.23–0.59 | <0.001 |

Multivariate logistic regression analysis was performed after adjusting for sex, age, witnessed status, place of cardiac arrest, bystander CPR, initial cardiac arrest rhythm, prehospital defibrillation, response time, scene time, transport time, and prehospital ROSC.

Abbreviations: aOR, adjusted odds ratio; CI, confidence interval; BLS, basic life support; CPR, cardiopulmonary resuscitation; ROSC, return of spontaneous circulation.

**Supplementary Table 5.** Initial vital signs and mental status those who were successfully resuscitated.

|  | Overall | BLS only | BLS  + advanced airway | BLS  + advanced airway  + epinephrine | P- value |
| --- | --- | --- | --- | --- | --- |
| **Initial vital signs** | n = 2124 | n = 319 | n = 1422 | n = 383 |  |
| Systolic Blood Pressure, mmHg | 116 [87–147] | 124 [100–150] | 116 [86–148] | 110 [80–138] | <0.001  ^a,b,c^ |
| Diastolic Blood Pressure, mmHg | 70 [52–90] | 77 [66–95] | 70 [52–90] | 66 [50–85] | <0.001  ^a,b,c^ |
| Systolic Blood Pressure <90mmHg, n (%) | 561 (26.4%) | 47 (14.7%) | 383 (26.9%) | 131 (34.2%) | <0.001  ^b,c^ |
| Heart Rate, /minute | 98 [76–120] | 98 [80–117] | 100 [78–121] | 93 [72–117] | 0.033  ^c^ |
| **Mental status and neurologic examination** |  |  |  |  |  |
| Glasgow Coma Scale* | 3 [3–7] | 10 [3–15] | 3 [3–7] | 3 [3–4] | <0.001  ^a,b,c^ |
| Glasgow Coma Scale <8, n (%)* | 998 (81.7%) | 74 (46.2%) | 704 (85.3%) | 220 (93.2%) | <0.001  ^a,b,c^ |
| Pupillary Light Reflex, n (%)** | 302 (43.2%) | 68 (75.6%) | 206 (43.1%) | 28 (21.4%) | <0.001  ^a,b,c^ |
| Corneal Reflex, n (%)** | 269 (38.5%) | 59 (65.6%) | 186 (38.9%) | 24 (18.3%) | <0.001  ^a,b,c^ |
| Self-Respiration, n (%)** | 294 (42.1%) | 68 (75.6%) | 191 (40.0%) | 35 (26.7%) | <0.001  ^a,b^ |

Data are expressed as median [interquartile range] or number (percentage) as appropriate.

^a^ Significant difference between the BLS only and BLS+advanced airway groups after Bonferroni correction in post hoc analysis.

^b^ Significant difference between the BLS only and BLS+advanced airway+epinephrine groups after Bonferroni correction in post-hoc analysis

^c^ Significant difference between the BLS+advanced airway and BLS+advanced airway+epinephrine groups after Bonferroni correction in post hoc analysis

* Glasgow Coma Scale was evaluated in 1221 patients those who were successfully resuscitated (n=160 for BLS only, n=825 for BLS+advanced airway group, n=236 for BLS+advanced airway+epinephrine groups).

** Pupillary light reflex, Corneal reflex, and self-respiration was evaluated in 699 patients those who were successfully resuscitated (n=90 for BLS only, n=478 for BLS+advanced airway group, n=131 for BLS+advanced airway+epinephrine groups).
